# Sodium acetate regulates milk fat synthesis through the activation of GPR41/GPR43 signaling pathway

**DOI:** 10.3389/fnut.2023.1098715

**Published:** 2023-02-16

**Authors:** Yingao Qi, Tenghui Zheng, Xinghong Liu, Siwang Yang, Qihui Li, Jiayuan Shao, Xiangfang Zeng, Wutai Guan, Shihai Zhang

**Affiliations:** ^1^Guangdong Province Key Laboratory of Animal Nutrition Control, College of Animal Science, South China Agricultural University, Guangzhou, China; ^2^State Key Laboratory of Animal Nutrition, Ministry of Agriculture and Rural Affairs Feed Industry Center, China Agricultural University, Beijing, China; ^3^College of Animal Science and National Engineering Research Center for Breeding Swine Industry, South China Agricultural University, Guangzhou, China; ^4^Guangdong Laboratory for Lingnan Modern Agriculture, South China Agricultural University, Guangzhou, China

**Keywords:** sodium acetate, milk fat, GPR41, GPR43, mTORC1, mammary epithelial cells

## Abstract

**Background:**

Fat is a critical component in milk, which provided energy for the early growth and development of mammals. Milk fat is positively related to the concentration of acetate in the blood, while the underlying mechanism is still unclear.

**Objective:**

This study is to investigate the effects of sodium acetate (NaAc) on milk fat synthesis in the mammary gland, and explored the underlying mechanism.

**Methods:**

*In vitro* experiments were carried out in mouse mammary epithelial cell line (HC11) cells cultured with NaAc to explore the potential pathway of NaAc on milk fat synthesis. Furthermore, 24 pregnant mice (from d 18.5 of gestation to d 7 of lactation, exposed to 200 mM NaAc drinking water) were used as an *in vivo* model to verify the results.

**Results:**

In this study, we found that NaAc promoted milk fat synthesis and the expression of related genes and proteins in HC11 mammary epithelial cells with the activation of GPCR and mTORC1 signaling pathways (*p* < 0.05). Pretreatment with the mTORC1 inhibitors and G protein inhibitors attenuated the NaAc-induced milk fat synthesis in HC11 mammary epithelial cells (*p* < 0.05). Importantly, the effect of NaAc on milk synthesis was attenuated in GPR41 and GPR43 knockdown HC11 mammary epithelial cells (*p* < 0.05). This evidence indicates that NaAc might regulate milk fat synthesis through the GPR41/GPR43-mTORC1 pathway. Consistently, in *in vivo* experiment, dietary supplementation with NaAc significantly increased milk fat content and fat synthesis-related proteins in mice mammary glands with the activation of mTORC1 and GPCR signaling pathways at peak lactation (*p* < 0.05).

**Conclusion:**

The addition of NaAc promoted the increase of milk fat synthesis in HC11 mammary epithelial cells and mice mammary glands at peak lactation. Mechanistically, NaAc activates GPR41 and GPR43 receptors, leading to the activation of the mTORC1 signaling pathway to promote the synthesis of milk fat.

**Figure:**
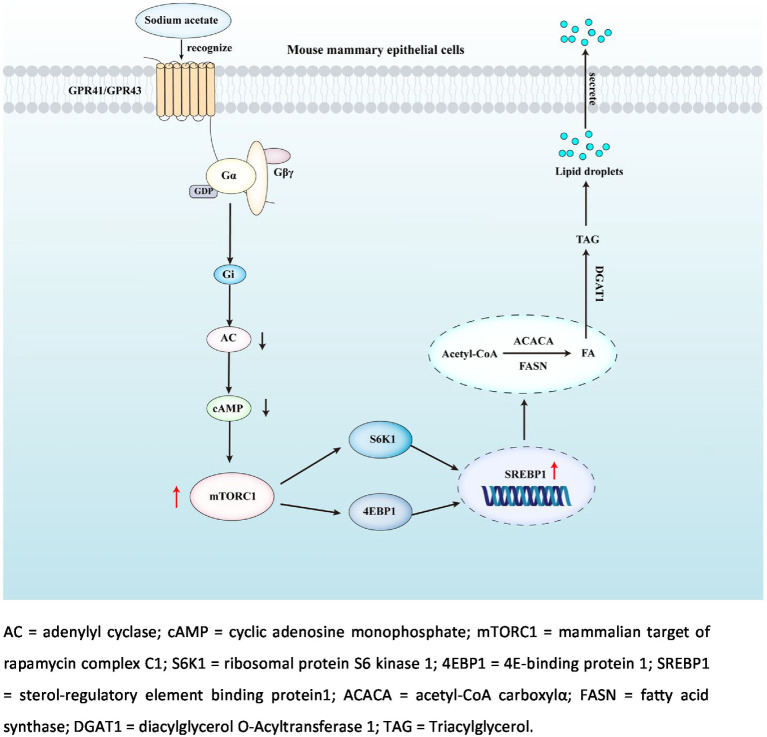
Graphical abstract

## Introduction

Milk is a critical nutrient source during mammalian offspring’s early life, which mainly includes fat, protein, lactose and small amounts of vitamins and minerals (1). Milk fat provided energy and essential fatty acids for neonatal growth and development, which is one of the most important components in milk (2). Milk fat could be affected by dietary energy and nutrients. Energy deficiency induces AMPK activation, which further inhibits milk synthesis by targeting PrlR and PGC-1α (3). Amino acids, such as branched-chain amino acids, methionine, arginine and lysine are also found to be involved in the regulation of milk synthesis in mammary epithelial cells (4). In addition, 18-Carbon fatty acids promote cytosolic triglycerides (TAG) accumulation by promoting genes and proteins related to milk synthesis (5).

In ruminants, short-chain fatty acids (SCFA) are largely produced by Rumen microbial fermentation of carbohydrates (6). High concentrations of SCFA not only act as energy substrates, but are also used to produce animal products, such as milk (7, 8). Compared with ruminants, monogastric animals are not able to efficiently digest dietary fiber, which leads to fewer SCFA in the stomach and the small intestine (9). Thus, due to the comparatively lower concentration of SCFA in monogastric animals, SCFA might not only act as a substrate but also function as a signaling molecule in the mammary gland.

G protein-coupled receptors (GPCR) are the largest family of cell membrane receptors, which are involved in the regulation of multiple cellular and physiological functions (10). GPCR contains 7 transmembrane protein structural domains which directly bind to G proteins in the membrane. G proteins are composed of three subunits, including G_α_ (bound to GTP or GDP), G_β_ and G_γ_ (11, 12). GPR41 and GPR43 have been identified as SCFA receptors in recent years. GPR43 initiates signal transduction by activating G_αi_/G_αq_ pathway, while GPR41 initiates signal transduction by activating the G_αi_ pathway (13, 14). One of the crucial downstream of GPCR is the mammalian target of rapamycin complex 1 (mTORC1). mTORC1 is a central coordinator of cell metabolism, which senses the nutritional status and regulates cell growth and physiological homeostasis (15–17). Importantly, mTORC1 promotes the generation of lipid droplets and the formation of TAG in mammary epithelial cells *via* regulating the activity of sterol regulatory element binding proteins (SREBP1), which is an important transcriptional regulator and nuclear receptor in *de novo* milk fat synthesis and changes in its mRNA abundance and protein expression affect milk fat synthesis (18, 19).

Previously, GPR41 and GPR43 have been reported to participate in the regulation of lipid metabolism (20, 21). However, it is still not clear the biological role of GPR41 and GPR43 in milk fat synthesis. Therefore, the primary objective of this study is to determine the role of GPR41 and GPR43 agonists (SCFA) on milk synthesis and identify the potential signaling pathway involved in this process. Understanding the mechanism of nutrients to promote milk fat synthesis is crucial to increase milk quality and beneficial to the health and development of neonates.

## Materials and methods

### Animals and experimental design

All of the procedures performed in animal feeding and sample harvesting during this study were approved by the South China Agricultural University Animal Care and Use Committee (no. 20110107-1, Guangzhou, China). Mice were fed a standard chow diet (provided by Guangdong Medical Experiment Center). And the major composition of raw materials is: Northeast corn, wheat, soybean meal, fish meal, chicken meal, multivitamins, vitamin K, vitamin E, vitamin D, animal fat, calcium hydrogen phosphate, trace elements, etc. As is shown in Supplementary Figure S1, mice were housed under a 12:12 light–dark cycle during the duration of study at 25°C and 50–60% humidity. Mice were provided *ad libitum* access to basal diet fed and fresh water. Twenty-four C57BL/6 mice (8 weeks old) were randomly divided into the control group and NaAc group and adapted 7–10 d. Male mice: female mice were mated in a ratio of 1:3. Treatment begins at d 18.5 of gestation to d 7 of lactation: The control group was fed with normal drinking water, and the treatment group was fed with water containing 200 mM NaAc (Sigma Aldrich, St. Louis, MO, United States). On d 7 of lactation, record the offspring’s weight, dam body weight and feed intake.

### Mammary gland sampling

On the morning of d 7 of lactation, 7 mice were euthanized after fasting for 12 h, and mammary gland tissue was collected using the method mentioned by Cheng et al. (22). Samples were quickly placed in liquid nitrogen after collection, and then stored in the −80°C refrigerator for testing.

### Milk sampling and analysis

On the morning of d 7 of lactation, mice’s milk was collected according to the method of DePeters et al. (23). Briefly, nursing pups were moved for 3 h, dams were ip-injected with oxytocin (4 IU per mouse) and anesthetized 5 min later with Avertin (Sigma Aldrich, St. Louis, MO, United States). Droplets of milk were aspirated by massaging the mammary gland and collected in a 2 mL siliconized microcentrifuge tube with the use of a vacuum system attached to a human breast pump (Evenflo, Miamisburg, OH, United States). Milk samples were stored at −20°C until analyzed. Two internal standards were used to determine the milk fat content (17:0 triacylglycerol and 19:0 FA methyl ester) (24). The content of prolactin in plasma was determined by ELISA (Huamei, Wuhan, China).

### Cell culture and treatment

HC11, mouse mammary gland cells, were cultured in complete medium (DMEM/F12 medium [Thermo]), mainly including 10% FBS (fetal bovine serum), 5 μg/mL IGF-1 (insulin-like growth factor-1) 10 ng/mL EGF (epidermal growth factor), 5 μg/mL ITS (Insulin-Transferrin-Selenium) and 10 μg/mL PS (penicillin–streptomycin) in cells incubator at 37°C and 5% CO_2_ concentration. To induce differentiation, cells were incubated in medium deprived of epidermal growth factor for 24 h and then cultured in DIP medium (1 μM dexamethasone, 5 μg/mL insulin, and 5 μg/mL prolactin) for the indicated time according to the experimental design. The reagents used in this experiment (sodium acetate, sodium propionate, and sodium butyrate and inhibitors) were provided by Sigma-Aldrich (St. Louis, MO, United States).

#### Experiment I

HC11 cells were cultured in different concentrations (0, 0.1, 0.75, and 1.5 mM) of NaAc, sodium propionate and sodium butyrate medium, *n* = 3. After 24 h incubation, HC11 cells were collected to examine lipid droplet formation with Oil red O staining, TAG content and the phosphorylation levels of ERK and CREB.

#### Experiment II

HC11 cells were cultured with different concentrations (0, 0.25, 0.5, 0.75, 1.0, and 1.5 mM) of NaAc, *n* = 3. After 24 h incubation, cells were collected to examine lipid droplet formation with Oil red O staining, TAG content and the genes and proteins expression of *FASN, ACACA, FABP3, DGAT, SREBP1* and the phosphorylation levels of mTOR, S6K1, and 4EBP1.

#### Experiment III

Pretreat HC11 cells with 50 nM Rapamycin for 2 h and then cells were cultured in 0.75 mM NaAc, *n* = 3. After 24 h incubation, cells were collected to examine lipid droplet formation with Oil red O staining, triglyceride content and the genes and proteins expression of *FASN*, *ACACA*, *FABP3*, *DGAT*, *SREBP1* and the phosphorylation levels of mTOR, S6K1, and 4EBP1.

#### Experiment IV

Pretreat HC11 cells with PTX, Gallein and NF023 for 2 h and then cells were cultured in 0.75 mM NaAc, *n* = 3. After 24 h incubation, cells were collected to examine proteins expression of FASN, ACACA, FABP3, DGAT, and SREBP1.

#### Experiment V

Pretreat HC11 cells with GPR41 siRNA and GPR43 siRNA (RiboBio, Guangzhou, China) for 6 h and then cells were cultured in 0.75 mM NaAc, *n* = 3. After 24 h incubation, cells were collected to examine TAG content.

### RNA isolation and real-time PCR

According to the instructions, total RNA was isolated from tissue and cell samples using an EZ-press RNA purification kit (EZ-Bio, Shanghai, China). The RNA samples were subjected to electrophoresis in agarose gel electrophoresis to verify their integrity. cDNA was prepared using an RNA reverse transcription kit (EZ-Bio, Shanghai, China) according to instructions. Real-time PCR was performed by ABI StepOnePlusTM real-time PCR Systems. The reaction system consists of 10 μL Real-Time PCR Master Mix, 2 μL cDNA, 0.8 μL of each PCR primer and 7.2 μL DEPC water. The following thermal profile was used for RT-PCR: 95°C for 1 min, followed by 40 cycles of denaturation at 95°C for 15 s, annealing at 59°C for 15 s, and extension at 72°C for 40 s. Set 40 repetitions per set. Relative gene expression was calculated according to the 2^−ΔΔCt^ method and β-actin was used to normalize the data. Primer sequences we used for real-time PCR are shown in Supplementary Table S1.

### Western blotting

Mammary tissue and HC11 cells samples were homogenized in RIPA lysis buffer supplemented with 1% PMSF. Total protein concentration was determined by BCA Protein Assay Kit (Biyuntian, Shanghai, China). Finally, the protein samples were boiled and denatured under 100°C and stored at −20°C for subsequent tests. The proteins were separated by electrophoresis on 10% or 12% SDS-PAGE gels and transferred to PVDF membranes (Sigma Aldrich, St. Louis, MO, United States). The membranes were blocked with 6% skimmed milk for 2 h after washing with Tris-buffered saline with Tween (TBST), followed by overnight incubation under gentle shaking with primary antibodies at 4°C. After washing with TBST, PVDF membranes were incubated with secondary antibodies for 1 h. The chemiluminescent signal was detected by using ECL reagents (P1020), and bands were quantified by ImageJ Software (ImageJ 1.52a). The antibodies used in this experiment are as follows. Primary antibodies: CREB (1:1000, 9197S) and P-CREB (1:1000, 9198S) purchased from Cell Signaling Technology (Danvers, MA, United States). FASN (1:2000, ab99359), ACACA (1:1000, ab72046), FABP3 (1:1000, ab231568), DGAT1 (1:500, ab100982), SREBP1 (1:1000, ab28481), 4EBP1 (1:1000, ab2606) and S6K1 (1:1000, ab9366) all purchased from Abcam (Cambridge, MA, United States); mTOR (1:1000, #2983), P-mTOR (1:1000, #5356), ERK (1:3000, 9102S), P-ERK (1:3000, 9101S), P-4EBP1 (1:1000, 9451S) and P-S6K1 (1:1000, #9234) all purchased from Cell Signaling Technology(Danvers, MA, United States). beta-actin (1:2000, bs-0061R) purchased from Beijing Bo Osen Biotechnology Co., Ltd. (Beijing, China); Secondary Antibodies: Goat anti-rabbit IgG (1:5000, 511,203) and Goat Anti-mouse IgG (1:5000, 511,103) purchased from ZenBio (Chengdu, China).

### Oil red O staining of lipid droplets and tag content analysis

The oil red O staining method was used to detect lipid droplets in HC11 cells. Cells were washed with PBS (Sigma Aldrich, St. Louis, MO, United States) for 3 times, then added with 4% paraformaldehyde and fixed at room temperature for 40 min. Subsequently, HC11 cells were washed twice with PBS, then added oil red O staining solution (Sangon Bio, Shanghai, China), and incubated at room temperature for 3 h. Discard the staining solution and rinse HC11 cells with 65% isopropanol once. Then HC11 cells were washed with PBS 5 times and observed under an inverted microscope (400×). In this study, Triglyceride Assay Kit (Jiancheng, Nanjing, China) was used to detect the content of TAG.

### Statistical analysis

Excel software was used to collect and sort out all data in this study, and SPSS 25.0 software was used for analysis. According to the experiment design, *T*-test and one-way ANOVA were used to analyze the results. All analysis results were expressed in the form of mean ± SEM, and *p* < 0.05 was used as the criterion of significance of difference.

## Results

### Short-chain fatty acids promoted the milk fat synthesis in HC11 cells

**Figure 1:**
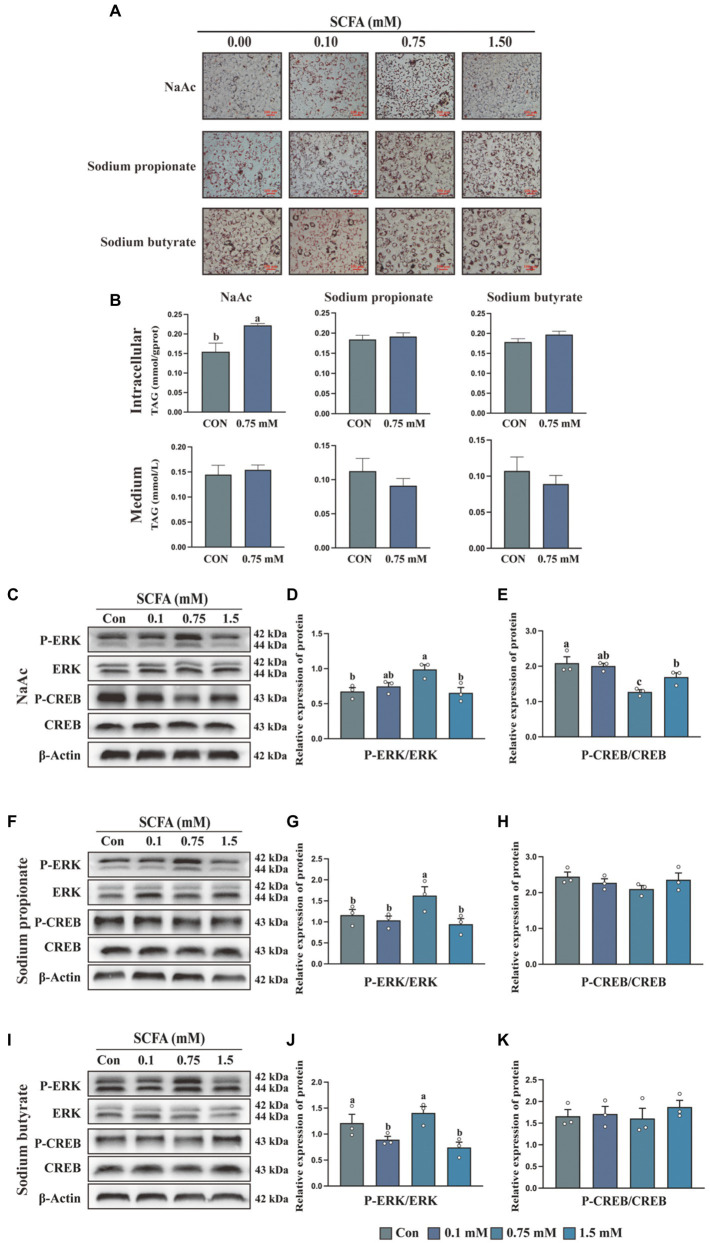
Short-chain fatty acids (SCFA) regulate milk fat synthesis in HC11 cells through the G_αi_ pathway **(A)** Oil red O staining images of HC11 cells treated with different concentrations of sodium acetate (NaAc), sodium propionate and sodium butyrate. Scale bars are 100 μm. **(B)** TAG content in intracellular and medium. The relative protein expression level of P-ERK/ERK and P-CREB/CREB were treated with different concentrations of NaAc **(C–E)**, sodium propionate **(F–H)**, and sodium butyrate **(I–K)**. All data with error bars are averages ± SEM (*n* = 3). In histograms, no letter or the same letter above the bar graph indicates no significant difference (*p* > 0.05), and different letters indicate a significant difference (*p* < 0.05). Each small white dot represents a test value.

To detect the effect of SCFA on milk fat synthesis, HC11 cells were treated with different concentrations of NaAc, sodium propionate and sodium butyrate. As shown in Figure 1A, compared with sodium propionate and sodium butyrate, NaAc efficiently promotes the synthesis of cells’ lipid droplets. The most effective concentration of NaAc on milk synthesis in HC11 cells is 0.75 mM, which significantly increases TAG content (Figure 1B; *p* < 0.05). While 0.75 mM sodium propionate and sodium butyrate had no significant effect on TAG synthesis in HC11 cells (Figure 1B).

### Differential regulation of ERK and CREB phosphorylation by short-chain fatty acids

Activation of the GPCR signaling pathway is characterized by the stimulation of the ERK/MAPK cascade (25). To test whether SCFA regulates milk fat synthesis through the GPCR signaling pathway, we detected the phosphorylation of ERK in HC11 cells treated with different concentrations of SCFA. NaAc (Figures 1C,D), sodium propionate (Figures 1F,G), but not sodium butyrate (Figures 1I,J) significantly increased the phosphorylation level of ERK at the concentration of 0.75 mM (*p* < 0.05). Previously study found GPCR-regulated cellular cAMP involved in the modification of mTORC1 activity, which is a crucial pathway for milk synthesis (26). Activation of G_αi_ protein could inhibit AC (adenylate cyclase) from converting ATP into cAMP, then inhibit the phosphorylation of CREB. Thus, the phosphorylation level of CREB is used as an indicator of G_i_ activation. As shown in the results, 0.75 mM NaAc (Figures 1C,E) significantly inhibited the phosphorylation level of CREB (*p* < 0.05), while sodium propionate (Figures 1F,H) and sodium butyrate (Figures 1I,K) had no effect on CREB phosphorylation. Thus, NaAc is selected for subsequent research.

### Sodium acetate promotes milk fat synthesis in HC11 cells with the activation of the mTORC1 signaling pathway

**Figure 2:**
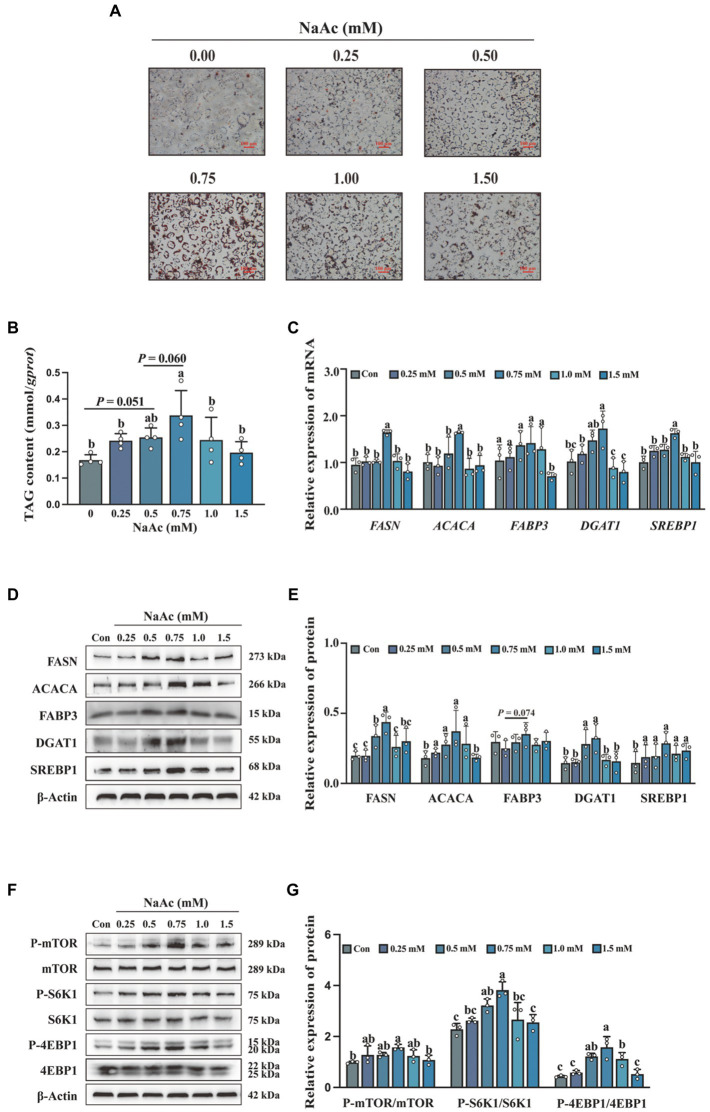
Sodium acetate (NaAc) promoted milk fat synthesis and activated the mTORC1 signaling pathway in HC11 cells. HC11 cells were exposed to different concentrations (0.00, 0.25, 0.50, 0.75, 1.00, and 1.50 mM) of NaAc. **(A)** Oil red O staining images of HC11 cells. Scale bars are 100 μm. **(B)** TAG content of HC11 cells. **(C)** Relative mRNA expression abundance of genes related to milk fat synthesis. **(D–G)** Relative expression abundance of proteins related to milk fat synthesis and mTORC1 pathway. All data with error bars are averages ± SEM (*n* = 3). In histograms, no letter or the same letter above the bar graph indicates no significant difference (*p* > 0.05), and different letters indicate a significant difference (*p* < 0.05). Each small white dot represents a test value.

To detect the effects of NaAc on milk fat synthesis, HC11 cells were treated with various concentrations of NaAc (0, 0.25, 0.5, 0.75, 1.0, and 1.5 mM) for 24 h. 0.75 mM NaAc significantly promoted lipid droplet formation (Figure 2A) and synthesis of TAG content (Figure 2B) in HC11 cells (*p* < 0.05). Consistently, 0.75 mM NaAc also up-regulated the expression of milk fat synthesis-related genes (*FASN, ACACA, DGAT1*, and *SREBP1*; Figure 2C) and proteins (FASN, ACACA, DGAT1, and SREBP1; Figures 2D,E) in HC11 cells (*p* < 0.05). In addition, NaAc activated the mTORC1 signaling pathway (as indicated by phosphorylation of mTOR, S6K1, and 4EBP1) in HC11 cells (Figures 2F,G; *p* < 0.05).

### The inhibition of the mTORC1 pathway in HC11 cells resulted in the weakened of sodium acetate-induced milk fat synthesis

**Figure 3:**
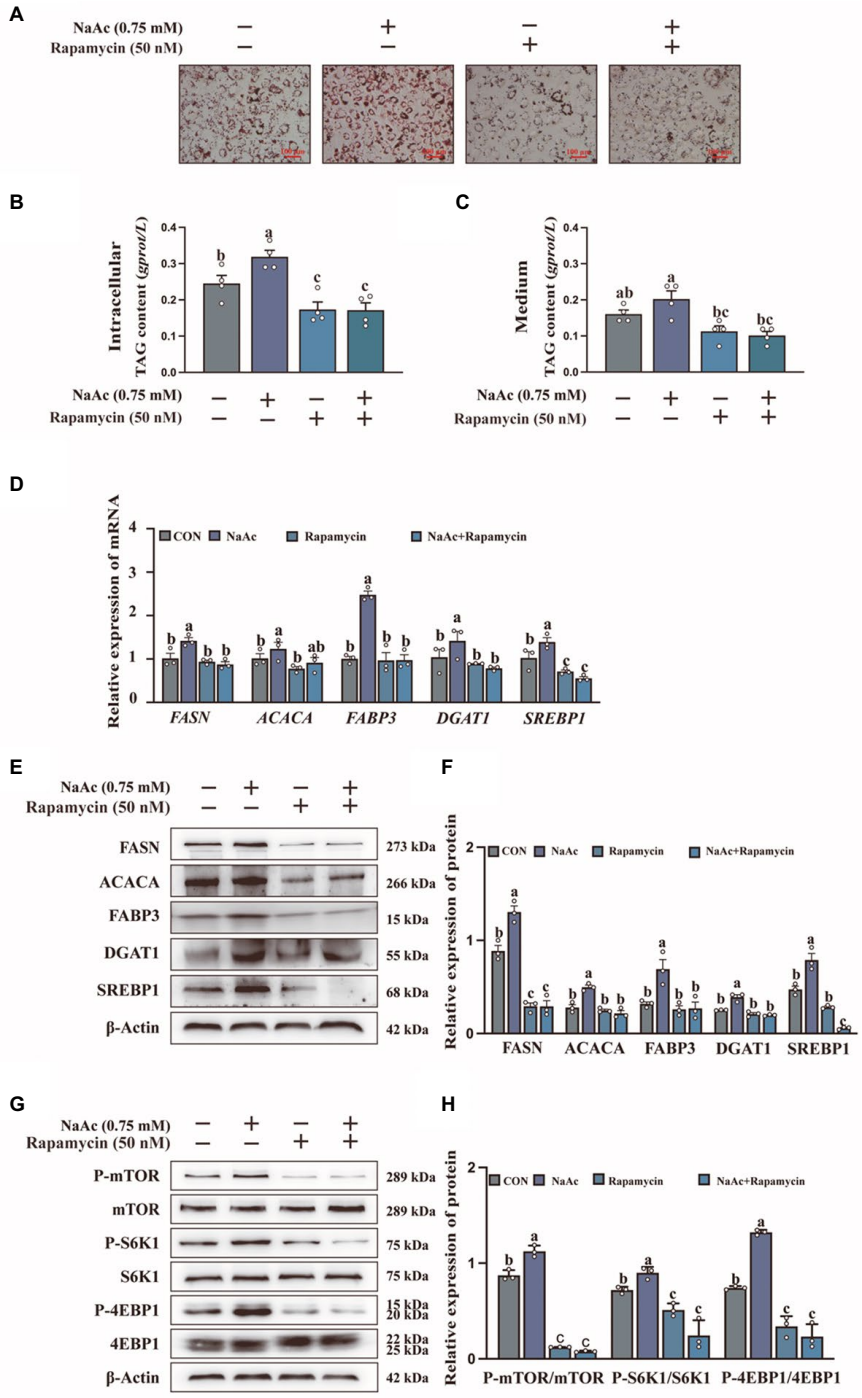
Effect of sodium acetate (NaAc) on milk fat synthesis and mTORC1 activation after pretreated with rapamycin. HC11 cells used in this figure were pre-treated for 2 h with 50 nM rapamycin (mTOR inhibitor) before incubation with 0.75 mM NaAc for 24 h. **(A)** Oil red O staining images of HC11 cells. Scale bars are 100 μm. TAG content in intracellular **(B)** and medium **(C)**. **(D)** Relative mRNA expression abundance of genes related to milk fat synthesis. **(E–H)** Relative expression abundance of proteins related to milk fat synthesis and mTORC1 pathway. All data with error bars are averages ± SEM (*n* = 3). In histograms, no letter or the same letter above the bar graph indicates no significant difference (*p* > 0.05), and different letters indicate a significant difference (*p* < 0.05). Each small white dot represents a test value.

To confirm the relationship between mTORC1 activation and milk fat synthesis, we analyzed the effect of NaAc on rapamycin pretreated HC11 cells. NaAc-induced lipid droplet formation (Figure 3A) and synthesis of TAG content (Figures 3B,C) were attenuated in rapamycin pretreated HC11 cells (*p* < 0.05). Consistently, mTORC1 inhibition also down-regulated the expression of milk fat synthesis-related genes (*FASN, ACACA, FABP3, DGAT1*, and *SREBP1*; Figure 3D) and proteins (FASN, ACACA, FABP3, DGAT1, and SREBP1; Figures 3E,F) induced by NaAc in HC11 cells (*p* < 0.05). As expected, rapamycin also inhibited NaAc-induced activation of mTORC1 signaling, as indicated by the phosphorylation levels of mTOR, S6K1, and 4EBP1 (Figures 3G,H; *p* < 0.05).

### Sodium acetate-induced milk fat synthesis is regulated through GPR41/GPR43-Gi pathways

**Figure 4:**
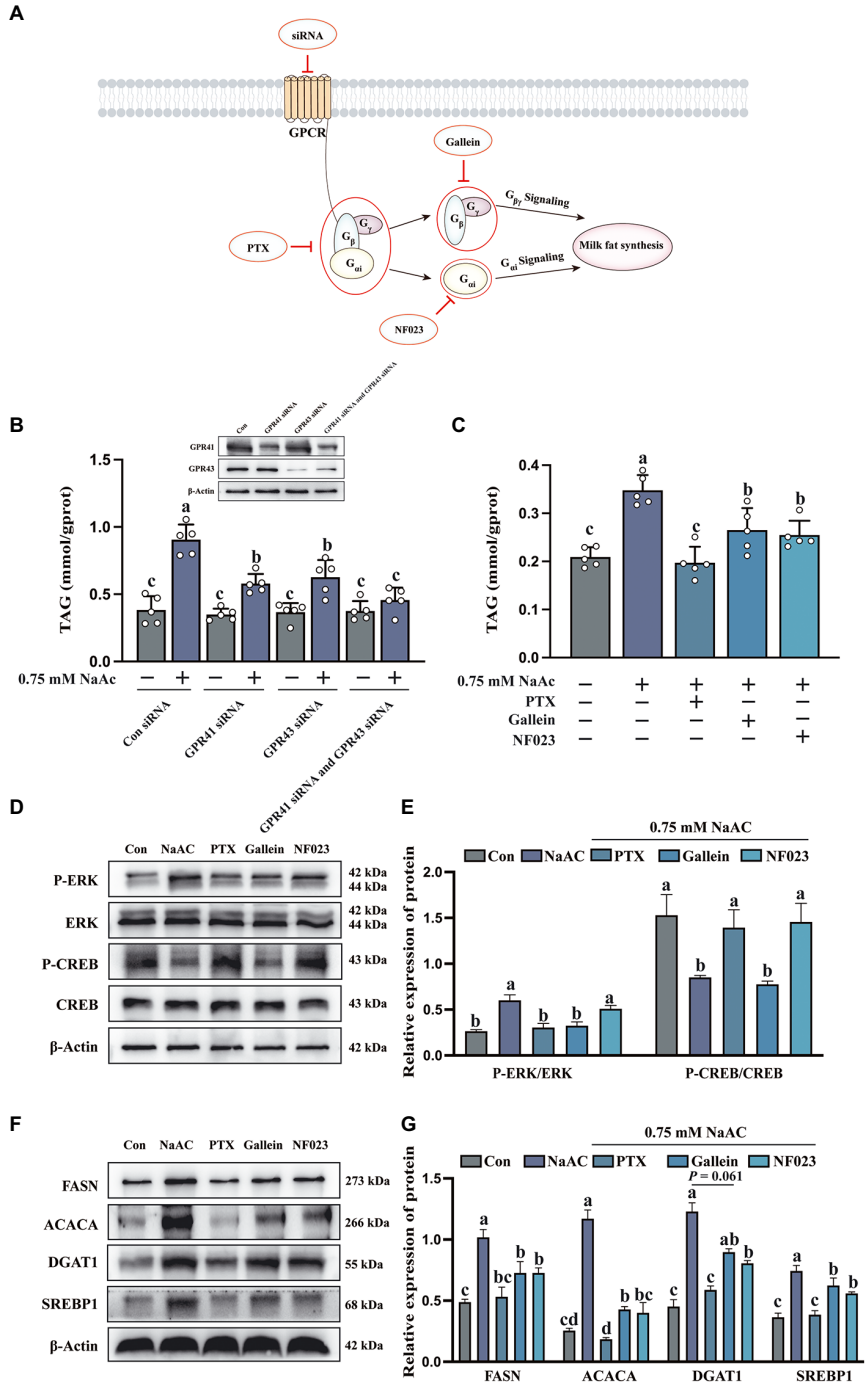
The inhibition of G protein attenuated the promoting effect of sodium acetate (NaAc) on milk fat synthesis. Cells used in this figure were pre-treated with PTX, Gallein, and NF023 for 2 h or GPR41 siRNA and GPR43 siRNA for 6 h, respectively, and then incubated with 0.75 mM NaAc for 24 h. **(A)** Targets of G protein-related inhibitors and siRNA. **(B,C)** TAG content in HC11 cells with different treatments, *n* = 5. **(D,E)** Phosphorylation degree of ERK and CREB in different treatments, *n* = 3. **(F,G)** Relative expression abundance of proteins related to milk fat synthesis, *n* = 3. All data with error bars are averages ± SEM. In histograms, no letter or the same letter above the bar graph indicates no significant difference (*p* > 0.05), and different letters indicate a significant difference (*p* < 0.05). Each small white dot represents a test value.

To further clarify whether GPR41 and GPR43 were involved in the regulation of NaAc-induced milk fat synthesis, a small interfering RNA (siRNA) knock-down approach was used to inhibit the expression of GPR41 and GPR43 in HC11 cells. As shown in Figure 4A, siRNA, PTX, NF023, and Gallein inhibited the GPCR receptors, G_i_, G_αi_, and G_βγ_, respectively. Intriguingly, NaAc-induced milk fat synthesis was significantly decreased both in GPR41 and GPR43 knockdown cells (Figure 4B; *p* < 0.05). This evidence indicated NaAc regulated milk synthesis through GPR41 and GPR43. To further clarify the downstream molecular mechanisms of GPR41 and GPR43, we used PTX, Gallein, and NF023, respectively. As shown in Figure 4C, PTX, Gallein, and NF023 treatments all inhibit NaAc-induced TAG synthesis in HC11 cells (*p* < 0.05). Furthermore, the effect of related inhibitors on G protein is shown in Figures 4D,E, according to the phosphorylation degree of ERK and CREB, PTX significantly inhibited G_i_, Gallein significantly inhibited G_βγ_, and NF023 significantly inhibited G_αi_ (*p* < 0.05). Consistently, NaAc-induced expression of the proteins related to milk fat synthesis (FASN, ACACA, DGAT, and SREBP1) was also significantly down-regulated when G_i_, G_βγ_, and G_αi_ were inhibited in HC11 cells (Figures 4F,G; *p* < 0.05).

### Sodium acetate regulates mouse offspring weight, milk fat content, G protein-coupled receptors and mTORC1 signaling pathways

**Figure 5:**
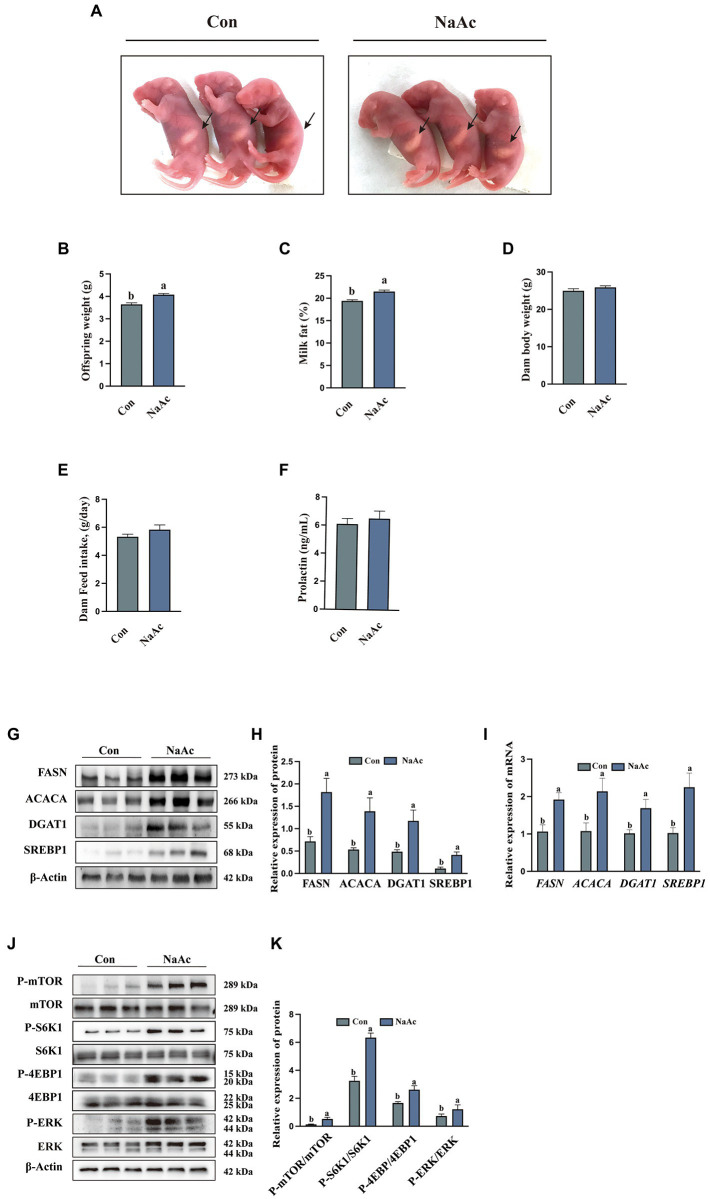
Effect of dietary supplementation of sodium acetate (NaAc) on milk fat synthesis and mTORC1 signaling in mice. **(A)** Milk in pups born by mothers, *n* = 3. **(B)** Offspring weight on d 7 of lactation, *n* = 7. **(C)** Milk fat content on d 7 of lactation, *n* = 7. **(D)** Dam baby weight on d 7 of lactation, *n* = 7. **(E)** Dam feed intake during lactation, *n* = 7. **(F)** Prolactin content in plasma of mice on d 7 of lactation, *n* = 7. **(G,H)** Relative expression abundance of proteins related to milk fat synthesis in mammary gland tissue, *n* = 3. **(I)** Relative mRNA expression abundance of target genes, *n* = 3. **(J,K)** Relative expression abundance of proteins related to GPCR and mTORC1 signaling pathways in mammary gland tissue, *n* = 3. All data with error bars are averages ± SEM. In histograms, no letter or the same letter above the bar graph indicates no significant difference (*p* > 0.05), and different letters indicate a significant difference (*p* < 0.05).

Dietary supplementation of NaAc significantly increased milk in pups born (Figure 5A) and offspring weight (Figure 5B) by mothers from NaAc treatment is observed, when compared to the control group (*p* < 0.05). Consistently, compared with the control group, dietary supplementation of NaAc significantly increased milk fat content (Figure 5C), but not dam body weight, dam feed intake and prolactin (Figures 5D–F). The expression of genes (*FASN, ACACA, DGAT1*, and *SREBP1*) and proteins (FASN, ACACA, DGAT1, and SREBP1) related to milk fat synthesis in the mammary gland tissues of mice were significantly increased in NaAc group (Figures 5G–I). In addition, NaAc supplementation activated the GPCR (judged by phosphorylation level of ERK) and mTORC1 (judged by phosphorylation levels of mTOR, S6K1, 4EBP1) signaling pathways (Figures 5J,K).

## Discussion

In recent years, the potential link between SCFA and milk fat synthesis has attracted wide attention (27). A large number of studies have shown that SCFA not only serves as signal molecules to promote fatty acid transport from adipose tissue to the mammary gland (28) but also directly participates in *de novo* milk fat synthesis as substrates (9). To date, the findings regarding SCFA on milk synthesis are mainly focused on ruminants, especially dairy cows. SCFA promoted the synthesis of intracellular TAG and significantly increased the mRNA abundance of genes related to milk fat synthesis (ACACA and FASN) and transcriptional regulation genes involved in lipid synthesis (SREBP1) in bovine mammary epithelial cells (BMEC) (7). Furthermore, SCFA also reduced the inflammatory response of BMEC by inhibiting histone deacetylase (29). However, the role of SCFA on the lactation performance of monogastric animals is not well elucidated. In this study, we found that NaAc efficiently promoted the lipid droplets in HC11 cells with the activation of GPCR and mTORC1 signaling pathways. Importantly, dietary NaAc supplementation enhances the milk fat content in lactating mice. However, it is worth noting that high concentration of acetic acid could be toxic to the cells and further inhibits milk fat synthesis (30).

GPR41 and GPR43 participated in the regulation of multiple biochemical and physiologic processes. For instance, activation of the GPR41/43 pathway inhibited the development of atherosclerosis (31). In addition, GPR41 activation resisted neuroinflammation, which could be used as a therapeutic strategy for treating Alzheimer’s disease (32). Importantly, both GPR41 and GPR43 are largely expressed in adipocytes and are involved in the regulation of lipid metabolism (33). High expression of GPR43, but not GPR41 promoted fat deposition (33, 34). The difference between GPR41 and GPR43 activation on fat synthesis might attribute to the difference of G proteins coupled to GPR41 and GPR43. GPR43 activates signal transduction by activating G_αi_/G_αq_ pathway, while GPR41 activates signal transduction by activating the G_αi_ pathway (13). Intriguingly, in this experiment, knockdown of either GPR41 or GPR43 by siRNA both decreased NaAc-induced TAG synthesis in HC11 cells, which indicated G_αi_ signaling pathway might play a vital role in this process. Activation of both GPR41 and GPR43 leads to increased intracellular Ca^2+^ concentration, activation of extracellular regulated protein kinase ERK1/2, and decreased cAMP concentration (35). When HC11 cells were treated with PTX (G_i_ inhibitor), Gallein (G_βγ_ inhibitor), and NF023 (G_αi_ inhibitor), the effect of NaAc on TAG synthesis was weakened, and PTX had the most obvious inhibitory effect on TAG synthesis and milk fat synthesis related protein. These results suggest that NaAc binding with GPR41 and GPR43 can promote the increase of milk fat synthesis in HC11 cells mainly through the activation of the downstream G_αi_ pathway.

mTORC1 is widely known as a critical regulator of protein synthesis through its downstream S6K1 and 4EBP1. Previous studies have pointed out that the mTORC1 signaling pathway is not only involved in the regulation of protein translation but also acted as an important regulator of lipid anabolism (36). In dairy cows, mTORC1 activation of regulates gene expression of key enzymes in mammary gland development and milk fat synthesis (37, 38). Furthermore, methionine can protect the mammary gland from oxidative stress by activating the mTORC1 pathway, thus promoting mammary gland development (39). In this experiment, NaAc-induced milk fat synthesis was significantly reduced when mTORC1 activity is inhibited by rapamycin. Recently, the relationship between GPCR and mTORC1 signaling are uncovered. Activation of G_αs_ could induce the cellular cAMP level which further inhibited the mTORC1 activity through PKA phosphorylation of raptor on ser 791 with the help of AKAP13 (40). Conversely, activation of G_αi_ could decrease the cellular level of cAMP, which might further promote the activation of mTORC1 (17). However, this hypothesis still needs further confirmation. In addition, it is still unclear how G_βγ_ regulates milk synthesis, which requires more future research.

## Conclusion

We have found that the addition of NaAc promoted the increase of milk fat synthesis in HC11 cells and mammary gland tissue of mice at peak lactation. Mechanistically, we believe that NaAc activated GPR41 and GPR43, leading to the activation of mTORC1 to promote the synthesis of milk fat.

## Data availability statement

The original contributions presented in the study are publicly available. This data can be found here: https://figshare.com/search?q=10.6084%2Fm9.figshare.22086329.

## Ethics statement

The animal study was reviewed and approved by South China Agricultural University Animal Care and Use Committee (no. 20110107-1, Guangzhou, China).

## Author contributions

YQ, SZ, XZ, and WG: designed the study. YQ, SZ, and JS: conducted research. YQ, SY, and QL: analyzed the data. YQ, TZ, and XL: wrote the manuscript. SZ: critically reviewed the manuscript. YQ had primary responsibility for the final content. All authors contributed to the article and approved the submitted version.

## Funding

This study was financially supported by the National Key R&D Program of China (2021YFD1300700), Guangdong Basic and Applied Basic Research Foundation (No. 2021A1515010440), Science and Technology Program of Guangzhou (No. 202102020056), and National Natural Science Foundation of China (Nos. 31802067 and 31872364).

## Conflict of interest

The authors declare that the research was conducted in the absence of any commercial or financial relationships that could be construed as a potential conflict of interest.

## Publisher’s note

All claims expressed in this article are solely those of the authors and do not necessarily represent those of their affiliated organizations, or those of the publisher, the editors and the reviewers. Any product that may be evaluated in this article, or claim that may be made by its manufacturer, is not guaranteed or endorsed by the publisher.

## Supplementary material

The Supplementary material for this article can be found online at: https://www.frontiersin.org/articles/10.3389/fnut.2023.1098715/full#supplementary-material
