# Supplementary material for: Sodium acetate regulates milk fat synthesis through the activation of GPR41/GPR43 signaling pathway
Source: Front Nutr. 2023 Feb 16;10:1098715. doi: 10.3389/fnut.2023.1098715 (PMC10035050; doi:10.3389/fnut.2023.1098715)
Supplement: Supplementary file 1 [file Data_Sheet_1.pdf]

**Supplementary Figure 1.** Graphical abstract of *in vivo* experiment

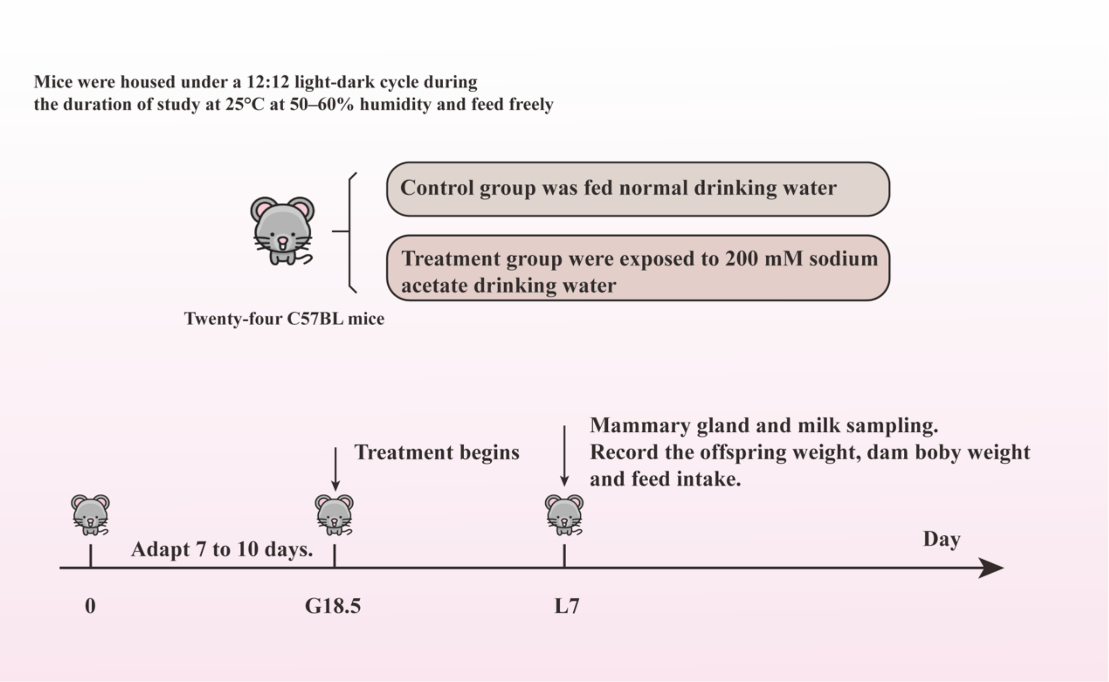

**Supplementary Figure 2.** Full original Western blot (WB) images for Fig. 1C, F and I

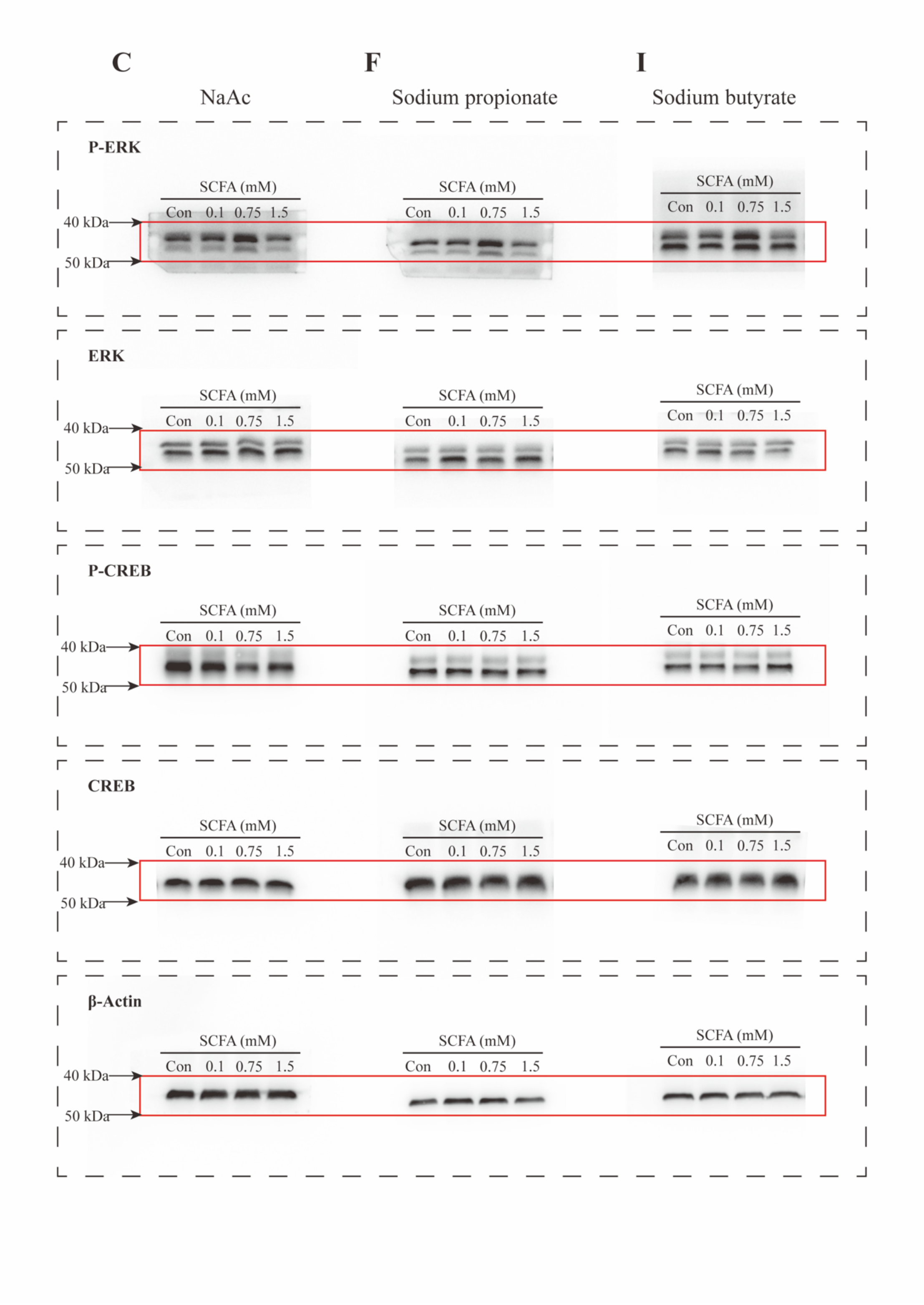

**Supplementary Figure 3.** Full original Western blot (WB) images for Fig. 2D and F

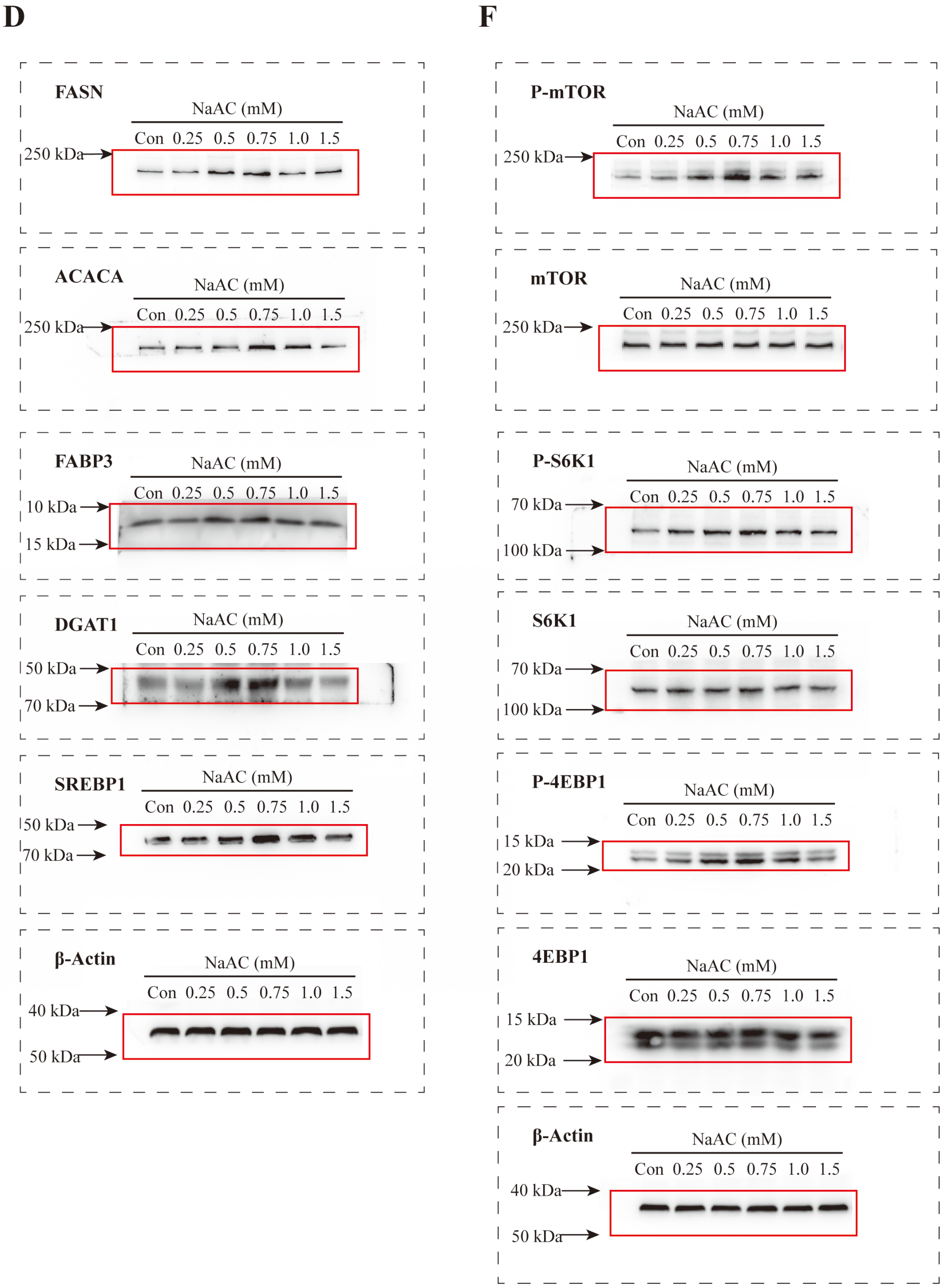

**Supplementary Figure 4.** Full original Western blot (WB) images for Fig. 3E and G

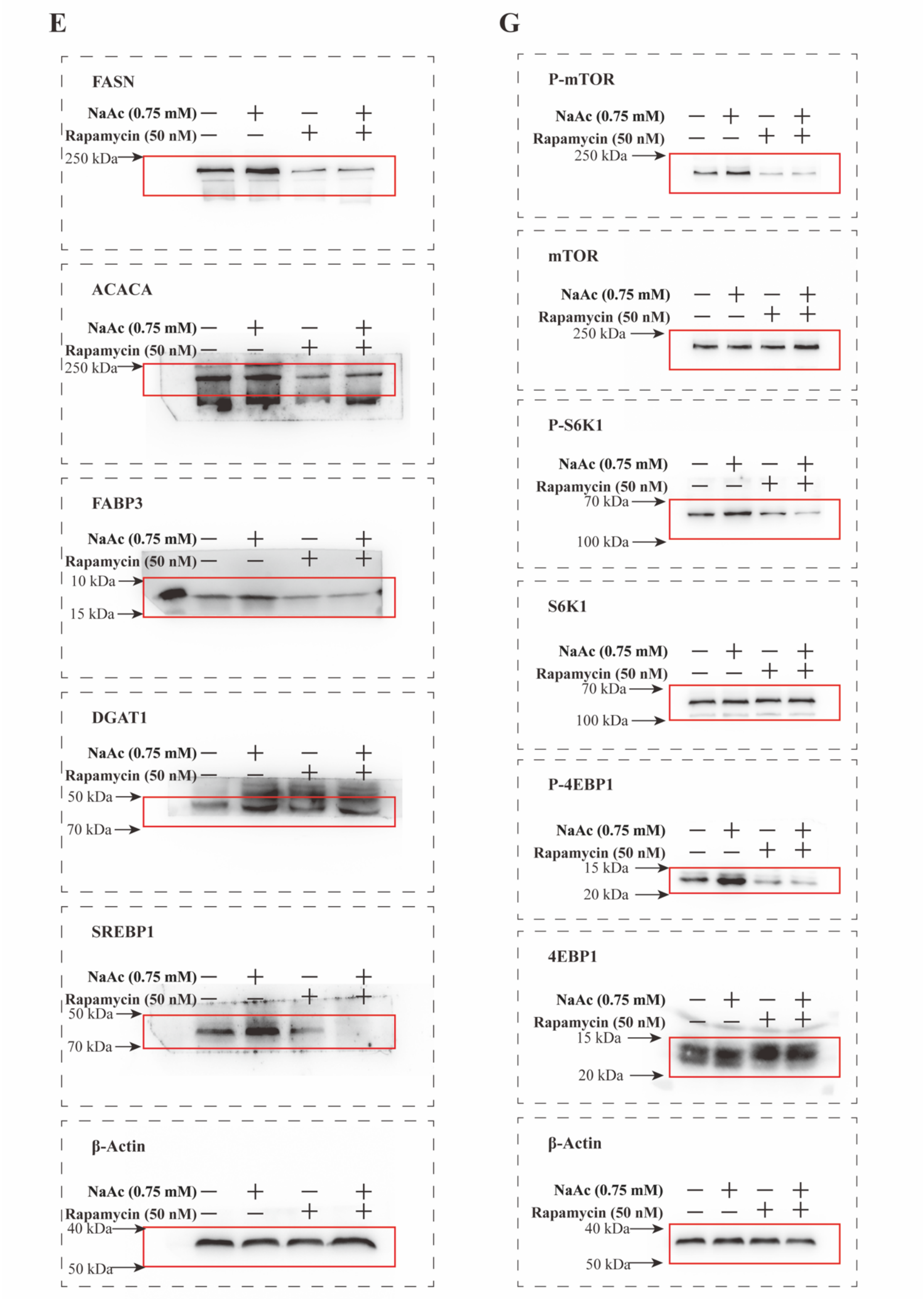

**Supplementary Figure 5.** Full original Western blot (WB) images for Fig. 4B, D and F.

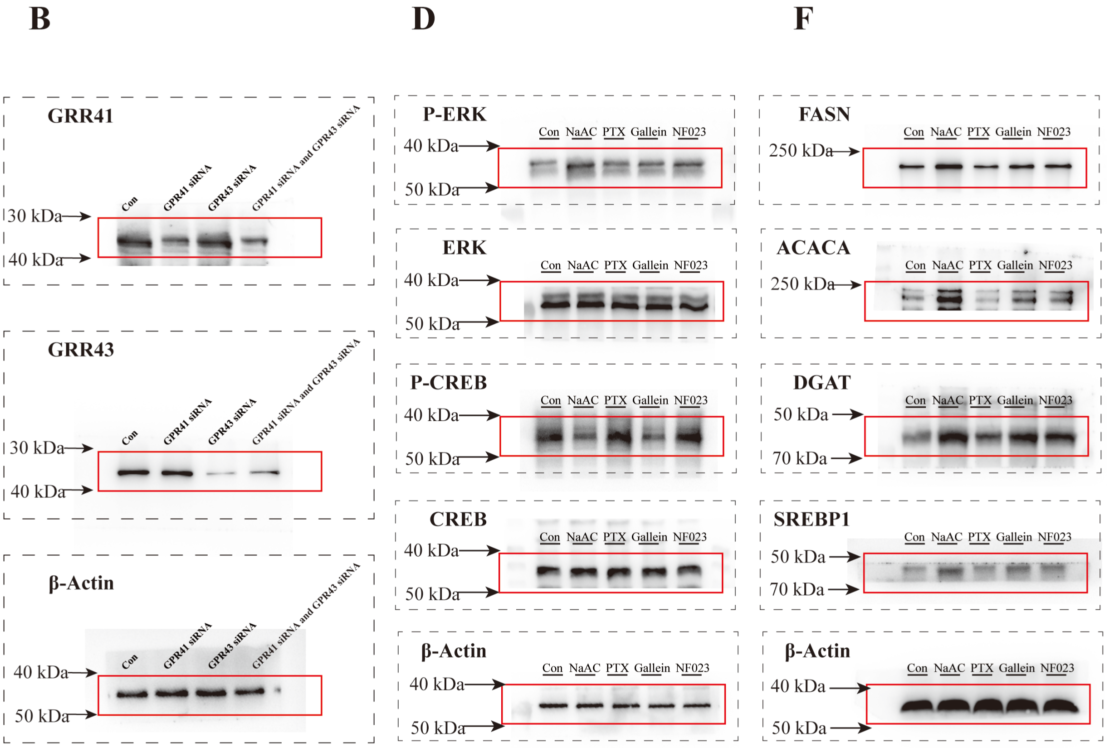

**Supplementary Figure 6.** Full original Western blot (WB) images for Fig. 5G and J

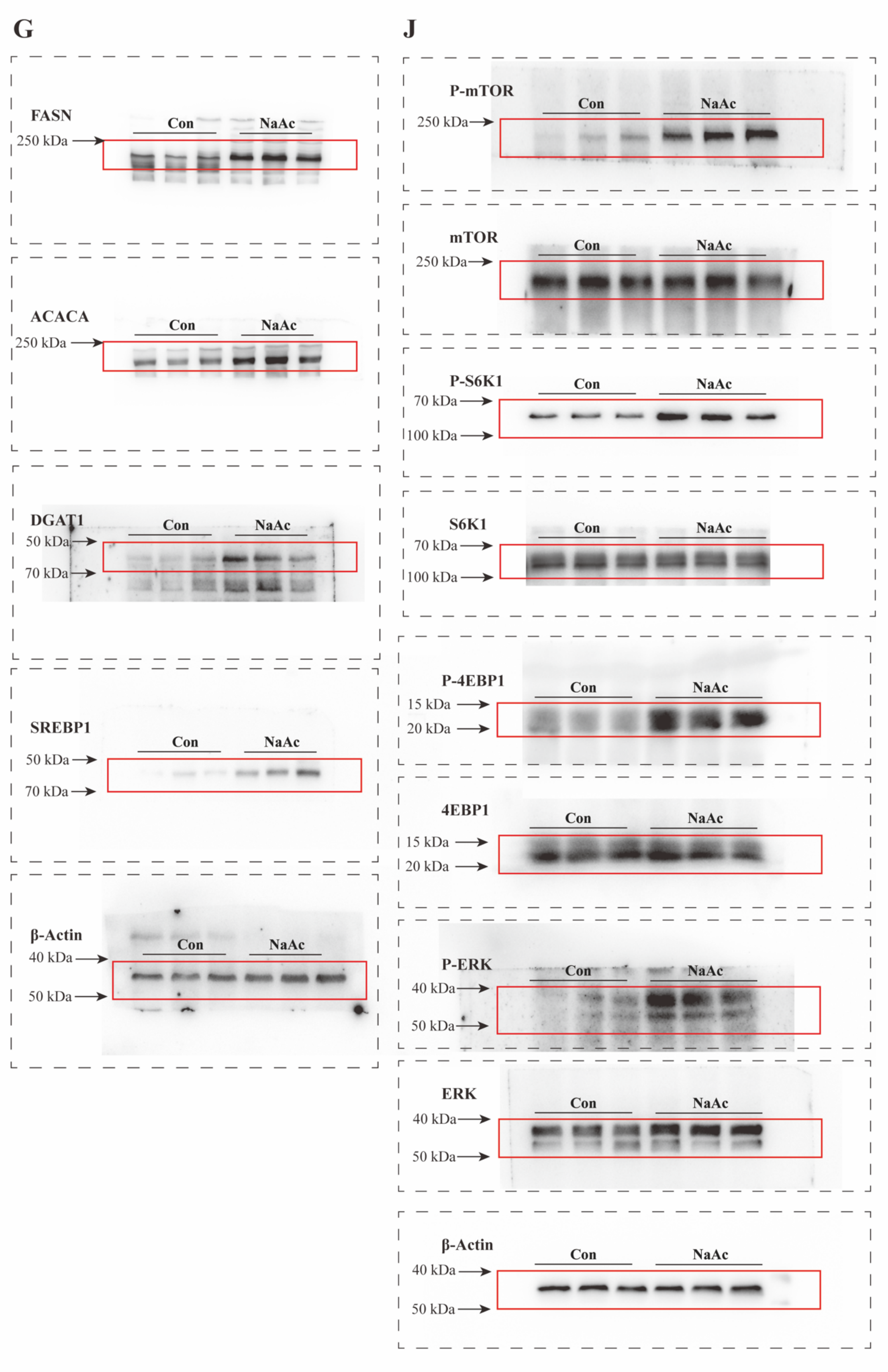

**Supplementary Table 1.** Primers used in this experiment

| Primers | Accession no. | Sequences (5′→3′) | Product size (bp) |
|---|---|---|---|
| *SREBP1* | XM_006532716.4 | F-AGAAGCTCAAGCAGGAGAACCTGA<br>R-ACTTCGGGTTTCATGCCCTCCATA | 127 |
| *ACACA* | XM_036156218.1 | F-GAAGTCAGAGCCACGGCACA<br>R-GGCAATCTCAGTTCAAGCCAGTC | 119 |
| *FASN* | NM_001099930.1 | F-GCTTGTCCTGGGAAGAGTGTA<br>R-AGGAACTCGGACATAGCGG | 115 |
| *FABP3* | NM_010174.2 | F-ACCTGGAAGCTAGTGGACAG<br>R-TGATGGTAGTAGGCTTGGTCAT | 106 |
| *DGAT1* | XM_006520405.4 | F-CAGCTGTGGCCTTACTGGTTGA<br>R-CGGCACCACAGGTTGACATC | 118 |
| *β-actin* | NM_007393.5 | F-CCACCATGTTACCCAGGCATT<br>R-CGGACTCATCGTACTCCTGC | 190 |

*SREBP1* = sterol-regulatory element binding protein1; *ACACA* = acetyl-CoA carboxylα; *FASN* = fatty acid synthase; *FABP3* = Fatty acid binding protein 3; *DGAT1* = diacylglycerol O-Acyltransferase 1.
